# Address to the Graduating Class of the Balto. College Dental Surgery

**Published:** 1881-04

**Authors:** William Kirkus


### ARTICLE III.

### *Address to the Graduating Class of the Baltimore College of Dental Surgery.* (Continued.)

DELIVERED BY REV. WILLIAM KIRKUS, M. A., LL. B.

It would almost certainly be impossible, it would perhaps be highly inexpedient, to attempt any such regulations as these in a country like the United States. Not only would it be impossible to secure uniform action through all the States of the Union, but there are here no ancient institutions commanding *universal* respect, like the English Universities and the great Medical Corporations, by which legislation might be guided. The advantages—without the drawbacks—of such legislative arrangements must be secured, among us, not by statute but by public opinion—and chiefly by the public opinion of the medical profession. And this is why I advise you never to forget that you are *physicians*. Never lower your profession either in your own persons or by encouraging low standards of medical education. In your own department of medical science and art, you occupy a position of very great advantage. Your college has a noble history and a high reputation. You need not be ashamed to be asked whence you come. Your predecessors have made a college which now can do very much toward making you. Not everything indeed —for one cannot "get a silk purse out of a sow's ear,"—but still very much. It will be for you to take care that you do not unmake your college—which were indeed a kind of parricide. There are colleges and there are colleges. When Bishop Tozer was in this country he was presented —so at least, I was gravely assured—with the diploma of Doctor of Divinity by what we should call in England a girls' school. I think he managed to evade accepting it. But take care that yours shall be not *a* college but *the* college of Dental Surgery in this broad new world. Show yourselves worthy of her. And use all your influence that her high standard of education shall be ever kept up to the topmost level of scientific research and mechanical skill.

I have urged upon you the importance of remembering that you are members of an illustrious and highly hon oured profession. May I venture to warn you not to be over-professional, nor to presume too much upon the ignorance of the general public even about the subjects of which you have so large and so just a monopoly. As a professional man never works only for money, and will never be bribed at any price to do inferior work, so he will recognize that he is a public instructor. One of the greatest services that physicians have rendered to the world in modern times has come from their persistent endeavours to render their own services unnecessary. Think how much is implied in such advice as this—you don't want a doctor but a plumber. Don't take medicine,—filter and boil the water you drink, and buy milk for a while at some other dairy. Unexplained causes, obscure and unintelligible diseases, are a continual vexation to the true physician, as they are a godsend to the quack. What in the world would a third-rate doctor do without malaria? Everything can be laid on those broad shoulders. Unskilful treatment fails; not of course because it is unskilful, but because the case was somehow complicated with malaria. Diptheria again, is a perfectly well-defined disease—but the adjective diptheritic is still one of the most serviceable in the dictionary. It is just as easy to grow small-pox as to grow cabbage or to breed pigeons—indeed it is unfortunately much easier; but I have myself heard a doctor (not in America) talk of an eruption as if it were both small-pox and something else—or neither—a sort of cross between the two—just as if one were to talk of an animal that was not exactly a dog and not exactly a cat but a kind of dog-cat. You can no doubt rely upon a vast amount of the stupidest and most inexcusable ignorance. Thousands of people think their stomachs are in their chests; and that their food 'settles' in the lungs; and have the queerest possible notions about their livers and their nerves and their blood. It is this ignorance upon which quacks practice. I suppose we all receive by mail, about every three weeks some nasty pamphlet about all kinds of diseases—and about new and secret medicines which this way or that way will infallibly cure them. Such trash would never be printed if there were not multitudes of people scandalously ignorant of the most rudimentary truths upon which their own physical comfort and sometimes even their life depends. And ignorant people always delight in sweeping generalities. This is the secret of the success of recent revolutionary and, so to speak, heretical systems of medicines: which are disproved not so much by the arguments of their opponents as by the practice of their professors. It is found that their high-sounding generalities will not work—and the new system has to revert in almost all severe cases which really require a positive and active treatment, to the old remedies. I do not, of course, say that this is dishonest; but surely a rule which has as many exceptions as examples is just a little premature.

Nothing has given me a more exalted opinion of the high honour both of the legal and medical profession than the extreme rarity of cases in which they have abused their enormous powers. The majority of mankind are absolutely powerless in your hands—they will do whatever you command, they believe almost anything you say. The more serious a disease is, the longer may be your attendance; and therefore, both directly and indirectly, the higher your remuneration. You might naturally suppose, therefore, that invalids would try to make out that their ailments were trifling. But we all know that this is the exact opposite of the truth. Many people—especially "lovely woman"—resent being supposed common-place sufferers. Children habitually exaggerate the maladies of their parents and of their brothers and sisters. Who is so mean as to have a sore throat which is less than diptheritic?

What baby would be worth its bottle that had anything less than a diptheritic croup? Everybody's sickness is one of the very worst cases the doctor ever knew. And yet, with this heavy premium on quackery, with this intense longing on the part of many of your patients that you would "fool them to the top of their bent," it is the rarest thing in the world to find a doctor who will condescend to disgrace himself by taking advantage of this peculiar frailty. Keep up gentlemen, this part of the dignity of your profession—and do more than keep it up. Try to make it impossible, by spreading light all around. Put down humbug, wherever you find it. Don't let your patients make fools of themselves. Be on your guard even against the dignity of the doctor, as clergymen should be on their guard against the dignity of their cloth. I do not mean, of course, the true dignity which should characterize everybody whose ordinary occupation brings him into incessant contact with the most solemn realities of life. But beware of make-believe dignity—affected mannerism—airs of infallibility—pomposity—mystery—and always be on your guard against the one man or woman in a hundred who really could find you out if you made yourself absurd. Remember that *the literature* even of your own peculiar profession is the common property of every intelligent reader. We of the clergy are sometimes told that popular science has taken away our occupation—stripped off our sacred vestments of mystery—and reduced us to the shivering unsophistication of two legged featherless bipeds. Well! I have no quarrel with science, popular or profound. I reverence and love it, and I honour its professors. If it beats us off the ground we surely deserve to be beaten—for here, as in so many other cases, might is right. But remember that popular science must put you on your mettle. The Johns Hopkins University doesn't give scientific lectures away without results. You will have forever hereafter, a far more appreciative but also a far more critical community to deal with. People won't go on accepting mere phrases instead of wisdom; and pompous mysterious airs instead of skill. In your department of life, as in mine, we may take to heart Tom Hood's words in his far too unfamiliar "Ode to Rae Wilson."

> "Mere verbiage! it is not worth a carrot!
> Why Socrates or Plato, where's the odds?
> Once taught a jay to supplicate the gods,
> And made a Polly-theist of a parrot!"

But not only will you be genuinely dignified, earnest, thorough, enthusiastic in your profession, but you can scarcely fail, as physicians, to acquire a certain reverent seriousness in the discharge of your duties and even in your ordinary behaviour and feeling. It is a very dreadful thing to stand so often between life and death; to grapple with that invisible foe, to whom sooner or later even your best skill must yield. No custom or familiarity, thank God, can ever so far brutralize you that you shall become indifferent to human suffering. Nor can you—least indeed of all men—fail to appreciate, if not the reasonableness, at any rate the immense power, of the religious emotions. I am told that a very large number of physicians are what people call infidels. If it be so—and I have no means of forming an accurate opinion—it would not I think be difficult to account for. But whatever your private opinion may be, you must constantly have to deal with people in those circumstances in which if they have any religion at all it is sure to make its appearance. It may be cowardly or heroic, it may be intelligent or superstitious, it may be soothing or exciting, it may stimulate or it may paralyse—but it will be there; and it will be there as a fact which no physician can dare to overlook. It will help or it may baffle all your skill. You cannot treat it with medicines; you cannot remove it by knife or cautery. You may say a wise word, you may check extravagance and reassure terror; but you will be able to do this only if you manifest at least an outward respect for the feelings which you may inwardly despise. And I

would venture to suggest that you will greatly benefit, if not yourselves at least your patients, if you can be persuaded to accept the alliance of those very persons whom you may sometimes be tempted to regard as your most dangerous foes—I mean the Christian clergy. I know that they are sometimes exceedingly injudicious—that they excite patients whose very life depends upon absolute repose. They weary the almost exhausted powers by fervid exhortation or long prayers. They sometimes seem to forget that killing the body is not the most obviously best mode of saving a soul. Nevertheless they will do you more good than harm,and you will find very many of them most useful assistants. I imagine it is the rule with most thoughtful clergymen—as it is most assuredly my own—to regard with the utmost respect the wishes and directions of the physician. And I *know* that whatever you may think of their spiritual efficacy, prayers and sacraments are often among the safest and most effective anodynes. You leave orders that the priest shall be excluded from the chamber of the sick or dying—and you will probably be obeyed. But you forget that the fearful heart of your patient may be all the while, in an agony of distress from which no treatment of yours can set him free. It is not you who can lift off the load of his sins, or

> "With some sweet oblivious antidote
> Cleanse the stuff'd bosom of that perilous stuff
> Which weighs upon the heart."

Ridiculous as you may think it, we *have* a power which you do not possess: and not seldom, though without a miracle, the sacraments of the church—some solemn confession, some soothing absolution, some holy viaticum, has healed the sick and brought back a departing soul from the very grasp of death. Is it not better that we should work together as friends? For you ought not to forget that if you refuse this alliance we shall certainly work as enemies. You need not suppose that we should ever give up our work or consent to regard it as subordinate.

We recognize the body as sacred—as the temple of the Holy Ghost. We cherish life as a divine gift, and a solemn trust from which no power in the world can release us. We do not believe that the salvation of the soul need to interfere with, but on the contrary promotes, the healing of the body. But if the alternative should really arise—not only in the case of voluntary martyrdom but in the ordinary course of nature—that either body or spirit must be sacrificed, we should not hesitate a moment. For my own part I believe that what are called death-bed repentances, are, at the most, so excessivaly rare that they cannot form the basis of an argument for practical life. I should not tease away the life of a dying man. On the contrary I should trust him to God, and when he had passed away, I should pray for his soul. Moreover publicans and sinners may often be nearer to heaven than Scribes and Pharisees. But in the last result we hold, every one of us, that if and whenever a man's spirit can be saved from baseness and defilement only by means which may imperil his health—his health must be imperilled. You may call this superstition. I call it rational religion and common sense. But anyhow it is a fact, and no merely material force has ever been able, in a close fight, to overcome spiritual and immaterial forces.

And even before we arrive at cases of extreme danger of death, there are cases of difference of opinion and treatment between doctors and divines which, I think, merits your serious attention—I mean cases of nervous disturbance and threatened or incipient insanity. I entirely believe that injudicious religious treatment has driven many people mad; and that cases of this kind become almost epidemic during religious revivals, and that this is a very serious set-off from their advantages. But none the less am I sure the medical treatment of such cases is often highly injudicious. I arrive at this conclusion from careful observation during a ministerial experience of thirty years. I have known many persons whose minds were being unhinged by religious excitement, advised (not to seek calmer counsel from more judicious spiritual guides, but) to divert their attention entirely from religion—to give up attending church, Bible reading and prayer. Sometimes the advice has been followed, and the unhappy patient has been lost to religion altogether—has been induced to do an outrage to conscience and to abdicate the soverign glory of human nature forever. But other and contrary results are exceedingly numerous. Outraged conscience resumes its sway. The old sins come back with the additional burden of a cowardly and wilful apostasy. There is a physical and hopeless relapse; and the well-meaning physician has killed both body and soul. Surely such disasters might be prevented by a better understanding between the clergy and the medical profession.

And now, gentlemen, in the words of Holy Scripture, "I wish you good luck in the name of the Lord." Be strong and show yourselves men. Work hard. Keep abreast of all knowledge. Remember your college, your Professors, your profession. And then you are sure to succeed. Or if not ,you will at least have the inestimable consolation of having deserved to succeed.

